# How the Intestinal Peptide Transporter PEPT-1 Contributes to an Obesity Phenotype in *Caenorhabditits elegans*

**DOI:** 10.1371/journal.pone.0006279

**Published:** 2009-07-21

**Authors:** Britta Spanier, Katrin Lasch, Silke Marsch, Jacqueline Benner, Wenjuan Liao, Hao Hu, Hermine Kienberger, Wolfgang Eisenreich, Hannelore Daniel

**Affiliations:** 1 Abteilung Biochemie, ZIEL Research Center of Nutrition and Food Sciences, Technische Universität München, Freising, Germany; 2 Abteilung Bioanalytik, ZIEL Research Center of Nutrition and Food Sciences, Technische Universität München, Freising, Germany; 3 Lehrstuhl für Biochemie, Technische Universität München, Garching, Germany; New Mexico State University, United States of America

## Abstract

**Background:**

Amino acid absorption in the form of di- and tripeptides is mediated by the intestinal proton-coupled peptide transporter PEPT-1 (formally OPT-2) in *Caenorhabditits elegans*. Transporter-deficient animals (*pept-1(lg601)*) show impaired growth, slowed postembryonal development and major changes in amino acid status.

**Principal Findings:**

Here we demonstrate that abolished intestinal peptide transport also leads to major metabolic alterations that culminate in a two fold increase in total body fat content. Feeding of *C. elegans* with [U-^13^C]-labelled *E. coli* revealed a decreased *de novo* synthesis of long-chain fatty acids in *pept-1(lg601)* and reduced levels of polyunsaturated fatty acids. mRNA profiling revealed increased transcript levels of enzymes/transporters needed for peroxisomal β-oxidation and decreased levels for those required for fatty acid synthesis, elongation and desaturation. As a prime and most fundamental process that may account for the increased fat content in *pept-1(lg601)* we identified a highly accelerated absorption of free fatty acids from the bacterial food in the intestine.

**Conclusions:**

The influx of free fatty acids into intestinal epithelial cells is strongly dependent on alterations in intracellular pH which is regulated by the interplay of PEPT-1 and the sodium-proton exchanger NHX-2. We here provide evidence for a central mechanism by which the PEPT-1/NHX-2 system strongly influences the *in vivo* fat content of *C. elegans*. Loss of PEPT-1 decreases intestinal proton influx leading to a higher uptake of free fatty acids with fat accumulation whereas loss of NHX-2 causes intracellular acidification by the PEPT-1 mediated proton/dipeptide symport with an almost abolished uptake of fatty acids and a lean phenotype.

## Introduction

Uptake of amino acids in form of di- and tripeptides by membrane transporters is a process found in all living organisms [1], [2]. Following peptide uptake into cells, amino acids are released by intracellular hydrolysis to serve as building blocks for protein synthesis, deliver precursors for more specialised synthesis or serve as fuel for ATP generation. In complex organisms peptide transporters are mainly found in epithelial cells in which they in addition to amino acids transporters contribute to overall supply with dietary amino acids [3], [4]. These mechanisms are also present in *C. elegans*. Numerous amino acid transporter systems are responsible for the uptake of free amino acids, while the uptake of di- and tripeptides is achieved by the intestinal low affinity/high capacity peptide transporter PEPT-1 (formally OPT-2, CPTB, PEP-2) [5], [6]. Peptide transporters are electrogenic proton-coupled symporters that utilize the transmembrane electrochemical proton gradient as driving force. Thus, substrate uptake depends strongly on membrane potential and extracellular pH [7]. Peptide-driven proton import leads to an intracellular acidification. Apical sodium-proton exchangers export protons back into the intestinal lumen followed by sodium ion import for regulation of the intracellular pH [8], [9]. The *C. elegans* PEPT-1 protein shows all characteristics of the mammalian intestinal peptide transporters [10], and similarly the Na^+^/H^+^ antiporter NHX-2 controls peptide-dependent pH_in_ homeostasis [11]. In *pept-1(lg601) C. elegans*, the deletion of the peptide transporter gene causes an amino acid deficiency, a reduced growth and a 60% decreased production of progeny when compared to wild type. A supplementation of pept-1*(lg601)* with free amino acids for compensation of the transporter defect increased the number of progeny by around 50% but had no effect on the retarded postembryonic development (∼80% compared with wild type) or the 30% reduced body size [5]. Therefore, a functional PEPT-1 transporter is critical for the amino acid homeostasis and its loss can not be completely compensated by the action of amino acid transporters. A loss-of-function of *nhx-2* leads to a similar phenotype as a loss of *pept-1*, indicating a strong interdependence of intestinal di- and tripeptide transport and Na^+^/H^+^ exchanger activity [11].

Most interestingly, in a full-genome RNAi screen, *pept-1* was found to be one of the major regulators of fat content in *C. elegans* [12], whereas animals deficient of the *nhx-2* gene displayed decreased lipid stores in intestinal cells [11]. Fat in the form of triglycerides is the main energy store in the nematode and it was found that triglycerides make up approximately 40–55% of total lipids in *C. elegans* [13]. The intestine is the major site of lipid storage where the triglycerides are deposited in fat granules [14]. Consequently, it is also the main organ for breakdown of stored TAG via lipolysis for peroxisomal and mitochondrial β-oxidation as well as for *de novo* synthesis of fatty acids from the precursor acetyl-CoA. In *C. elegans* like in mammals, insulin signaling participates in the control of fat metabolism and worms with a loss-of function of *daf-2*, the insulin-like receptor, have an increased body fat content and the induced metabolic shifts lead to a dauer larva-like metabolism and a higher stress resistance [12], [15], [16]. The increased fat content enables the worm to survive conditions of low food supply by utilizing the stored fat as main energy source. Although *pept-1* interacts with the *daf-2* insulin-like signaling pathway, it is important to note that the fat accumulation in *daf-2* is associated with dauer formation and metabolism which than also leads to an increased lifespan, while the amino acid deprivation caused by a loss of *pept-1* does not affect dauer and has no effect on lifespan [5].

Despite convincing evidence that the intestinal peptide transporter contributes to lipid storage and metabolism in *C. elegans* as derived from the RNAi screen, nothing is known about the underlying processes. We therefore systematically analysed which metabolic routes involved in lipid absorption, storage and break-down in *C. elegans* are altered in *pept-1(lg601)* mutants. For this purpose, we used a combination of bioanalytical methods, such as RNA and metabolite profiling, *in vivo* isotope labeling combined with NMR spectroscopy as well as RNA interference. We demonstrate that the lack of intestinal di- and tripeptide transport leads to a dramatic shift in gene expression of lipid metabolic pathways and show that the metabolic turn-over of free fatty acids and lipids is altered. Concentrations of short- and medium-chain fatty acids are highly increased in *pept-1* worms while the *de novo* synthesis of long-chain fatty acids and more so of polyunsaturated fatty acids is drastically decreased. We showed for the first time in an *in vivo* system, that the functional correlation of the pH_in_ of the intestinal epithelial cells via the peptide transporter PEPT-1 and the Na^+^/H^+^ exchanger NHX-2 is an essential regulator for the uptake of free fatty acids from the diet.

## Results

### Total body fat is increased in *pept-1(lg601)*

**Figure 1:**
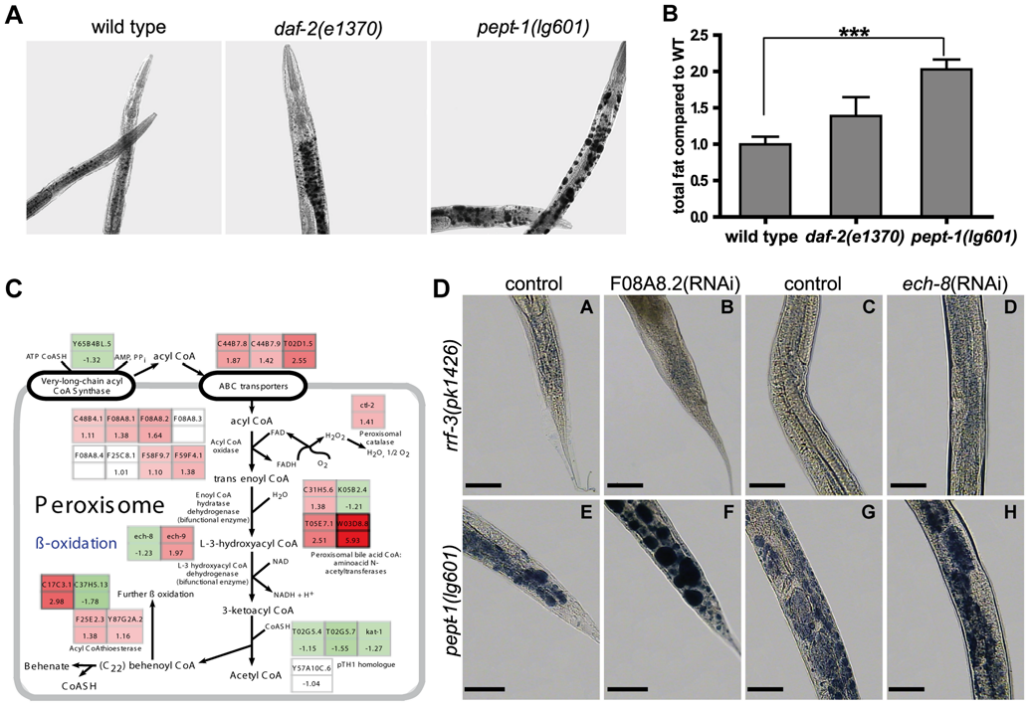
Body fat content in wild type, *daf-2(e1370)* and *pept-1(lg601) C. elegans*. (A) The worms were stained with Sudan Black to visualize the body fat. In each image, the anterior end is positioned to the top. (B) Total fat in comparison to wild type (set to one) was calculated based on gas chromatographic measurements. (C) Changes in mRNA expression of genes involved in peroxisomal β-oxidation of fatty acid metabolism in *pept-1(lg601) C. elegans*. The data are based on microarray analysis data in comparison to wild type (n = 5). Genes expressed in low levels in *pept-1(lg601)* are highlighted in green boxes, and genes expressed in higher levels are highlighted in red boxes. The darker the color, the higher is the change in expression. Genes with changes in expression<0.1 are not colored. (D) Impact of peroxisomal β-oxidation on the body fat content in *rrf-3(pk1426)* and *pept-1(lg601) C. elegans*. RNAi knockdown of F08A8.2 (coding for acyl CoA oxidase) had no effects on the body fat in *rrf-3* (A, B) but increased the total body fat in *pept-1(lg601)* leading to extremely large fat droplets in the posterior part of the worms (E, F). Knockdown of *ech-8* (coding for peroxisomal bifunctional enzyme hydroxyacyl-CoA dehydrogenase/enoyl-CoA hydratase) slightly increased the body fat in *rrf-3* when compared to control *rrf-3* (C, D). The effect was more pronounced in a *pept-1(lg601)* background (G, H). The body fat was detected by Sudan Black staining. Scales indicate 50 µm.

To visualize the total body fat of wild type, *daf-2(e1370)* and *pept-1(lg601)*, populations of these worm strains were stained after fixation with Sudan Black. For *daf-2 C. elegans* increased body fat has been shown and animals also possess increased phospholipid and glycerolipid concentrations compared to wild type [12]. We found that the *pept-1* animals accumulate an even higher amount of body fat than *daf-2* (Fig. 1A). Sudan Black staining indicated much larger fat granules in intestinal cells of *pept-1* than in wild type and in *daf-2* worms. Lipid extraction from L4 larvae revealed a 2.0±0.1 fold increased total body fat in *pept-1* over wild type (p<0.001), while *daf-2* showed an 1.4±0.3 fold increase in total body fat (Fig. 1B). These data are contrary to those reported by Ashrafi et al., 2003 showing that *pept-1* RNAi treated animals possessed a significantly decreased body fat content based on a Nile Red screen. To better understand the underlying mechanisms at the gene and metabolic level responsible for the increased fat accumulation, we analysed the mRNA expression on whole genome arrays and fatty acid metabolism by biochemical means in *pept-1(lg601)*.

### Fatty acid break-down in *pept-1*

Comparison of the mRNA expression patterns of *pept-1* and wild type L4 larvae using a microarray-based full-genome transciptomics approach revealed a change in expression of around 3000 genes. We identified 1643 genes with more than 1.5-fold up-regulation in expression level and 1355 genes with an at least 1.5-fold down-regulation in transcript levels in *pept-1* compared to wild type (Table S1 available online). Based on gene ontology (GO) over-represented pathways with the most prominent changes were “Lipid and Fatty Acid Metabolism”, “Cell Signaling” and “Amino Acid Metabolism”. For genes involved in fatty acid catabolism mRNA expression profiles revealed mainly a significant down-regulation. However, for peroxisomal proteins involved in fatty acid oxidation an up-regulation of transcripts in *pept-1(lg601)* was observed (Fig. 1C). We concentrated on these peroxisomal β-oxidation pathways and assessed their role in lipid accumulation in *pept-1(lg601)*.

The peroxisomes are central organelles for the break-down of very-long and long-chain fatty acids in eukaryotes. The peroxisomal β-oxidation generates H_2_O_2_, NADH and shortened acyl-CoA's as the major end products. Since H_2_O_2_ can potentially damage macromolecules its elimination by catalase, which is abundant in peroxisomes, is an important process. In *C. elegans* CTL-2 is the peroxisomal isoform and the transcriptome data revealed 1.4-fold increased mRNA-levels in *pept-1*. This was confirmed by a 2.5 fold higher catalase activity in *pept-1* than in wild type worms (data not shown). Among the other genes that showed increased expression levels in *pept-1* were the ABC transporters needed for acyl-CoA import into peroxisomes as well as almost all enzymes of the β-oxidation chain including the acyl-CoA oxidases and 3-ketoacyl-CoA thiolases. To functionally asses whether these changes contribute to the altered lipid storage capacity in *pept-1*, we employed RNA interference (RNAi) and silenced seven selected genes that on avarage showed increased mRNA levels (*pmp-4*, F08A8.2, T05E7.1, W03D8.8, *ech-8*, *ech-9*, C17C3.1) followed by measurements of fat accumulation. Only the knockdown of two genes revealed a significant change in body fat content without other servere phenotypic changes. Knockdown of F08A8.2 which encodes for an acyl CoA oxidase, and *ech-8* which encodes for the peroxisomal bifunctional enzyme (hydroxyacyl-CoA dehydrogenase/enoyl-CoA hydratase) significantly increased total body fat in *pept-1(lg601)* but not in *rrf-3 C. elegans* (Fig. 1D). While *ech-8* RNAi led to a higher number of small fat droplets, F08A8.2 RNAi induced a dramatic increase in the mean diameter of the fat droplets. These findings may be taken as an indicator that the on average observed increased expression of genes coding for peroxisomal proteins may indeed increase long-chain fatty acid breakdown in *pept-1*, as the knockdown of selected genes also lead to a further increase in body fat stores (Fig. 1C and D).

### *De novo* synthesis of fatty acids is decreased in *pept-1(lg601)*

**Figure 2:**
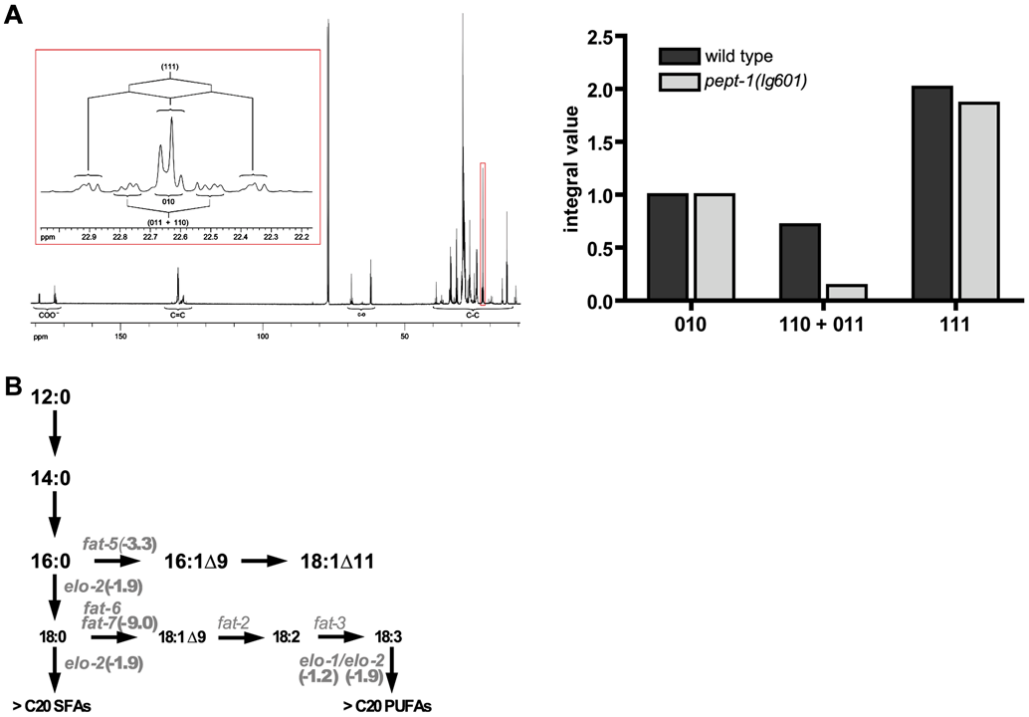
The *de novo* synthesis of fatty acids is reduced in *pept-1(lg601) C. elegans*. (A) *Left panel*, ^13^C NMR spectrum of a lipid extract from *C. elegans* grown on a 1∶10 mixture of [U-^13^C]-*E. coli* and unlabelled *E. coli*. The inset displays the signal marked by the red box in the full spectrum. ^13^C-^13^C coupling patterns are indicated and assigned to the isotopologues groups (010), (110+011), and (111), respectively. *Right panel*, Relative ^13^C-NMR intensities for ^13^C_1_ [010], ^13^C_2_ [110, 011] and U-^13^C [111] fatty acids, as determined from the coupling pattern of a methylene carbon atom group resonating at 22.6 ppm in wild type and *pept-1(lg601)*. The low signal contribution of the [110, 011] group in *pept-1* indicates a low *de novo* synthesis of long-chain fatty acids in this worm strain. (B) Fatty acid elongation and desaturation in *pept-1(lg601)* compared to wild type (scheme based on Brock et al., 2007). Short-chain saturated and medium-chain monounsaturated fatty acids accumulate (bold) at the expense of long-chain polyunsaturated fatty acids (thin). The effect is based on a down-regulation of most genes coding for elongases and desaturases that are active in the synthesis of PUFAs. mRNA expression levels compared to wild type are added in brackets.

To assess functionally whether *de novo* fatty acid synthesis is altered in *pept-1* as suggested by the mRNA expression data, we used an NMR based isotopologe profiling approach. Lipid extracts of wild type and *pept-1 C. elegans* grown on a 1∶10 mixture of [U-^13^C]-labelled and unlabelled *E. coli* were analysed. Specifically, the well-resolved ^13^C-NMR signal of methylene carbon atoms resonating around 22.6 ppm displayed satellite pairs due to simultaneous coupling with two ^13^C-neighbours which were distinguished from pairs due to a single ^13^C-coupling (Fig. 2A). On this basis, ^13^C-isotopologues of fatty acids comprising different numbers of ^13^C-atoms were identified and quantified (Fig. 2A). The sum of [110] and [011] can be taken as a quantitative measure for *de novo* fatty acid synthesis. Due to the fact that the signal group at 22.6 ppm can be assigned tentatively to ω-2 CH_2_ atoms in long-chain saturated or ω-6 unsaturated FAs, the relative decrease of [110] and [011] in the *pept-1* line indicates a markedly reduced *de novo* synthesis rate of around 30% of that in wild type (3.9 versus 8.9 weight%) of these FAs. These findings correlate well with the fatty acid profiling results and the transcriptome data that revealed a down-regulation of most genes coding for proteins in *de novo* fatty acid and lipid synthesis pathways (Tabel S1 available online). The availability of dietary fatty acids from *E. coli* OP50 and their use for endogenous chain elongation was recently reported based on a stable isotope assay [17]. According to these findings almost all saturated fatty acids of >C16 found in worms are derived from the diet (>90%) which can undergo further endogenous chain elongation and desaturation after intake, whereas the odd-numbered C15 and C17 fatty acids are completely synthesized *de novo* in worms from the branched chain amino acid precursors. Based on these observations we also performed a fatty acid analysis in *pept-1* by GC-MS (Table 1).

**Table 1:** Fatty acid composition of wild type and *pept-1(lg601)*.

Fatty acid	Wild type	*pept-1*
C12:0	---	0.31±0.09
C14:0	1.17±0.05	1.49±0.1
C16:0	3.33±0.11	4.58±0.37**
C18:0	4.17±0.06	3.39±0.21**
C20:0	0.56±0.01	0.39±0.01
C22:0	0.78±0.01	0.60±0.01
Total SFA	9.9	10.7
C16:1	1.95±0.01	5.06±0.16***
C18:1 Δ9	3.03±0.04	3.33±0.08**
C18:1 Δ11	15.0±0.08	21.5±0.58***
Total MUFA	20	29.9
C18:2	7.82±0.03	5.12±0.13***
C20:2	1.97±0.02	0.73±0.04***
C20:3	0.72±0.01	0.35±0.01***
C20:4	4.19±0.02	2.32±0.05***
C20:5	8.12±0.05	4.20±0.47***
Total PUFA	22.8	12.7
C17:0	16.20±0.08	16.28±0.15
C19:0	14.73±0.06	7.66±0.20***
iso-C15:0	3.07±0.02	1.67±0.04***
iso-C17:0	4.29±0.02	1.27±0.02***

Data are weight percentages (mean±SD) of three independent trials of total worm fatty acids measured by gas chromatography. Normalization was performed by taking the sum of the peak areas of all detected fatty acid in a chromatogram as 100%. Dashes indicate fatty acids with weight% lower than 0.3%. Significant differences to wild type were determined using the students t-test (*: p<0.05, **: p<0.01, ***: p<0.0001). SFA, saturated fatty acid; MUFA, monounsaturated fatty acid; PUFA, polyunsaturated fatty acid; Δ, cyclopropane fatty acid.

### *pept-1* show major changes in fatty acid profiles

Fatty acid analysis in peptide transporter deficient worms revealed large changes in individual classes of fatty acids when compared to wild type (Table 1). Although the content (weight percent) of saturated fatty acids was the same as in wild type, *pept-1* showed a higher concentration of C16:0 which serves as precursor for further chain elongation and desaturation steps and is in worms exclusively derived from the bacterial food [17], [18]. The fraction of monounsaturated fatty acids (MUFA) was increased by about 50% in *pept-1* in expense of the polyunsaturated fatty acids (PUFA) which were decreased by 45% on average (Table 1). This corresponds well with the results from our mRNA expression analysis, where genes coding for the elongases and desaturases needed in PUFA synthesis are mostly down-regulated in *pept-1* (Fig. 2B) which may translate into a decreased capacity for synthesis of saturated long-chain and all polyunsaturated fatty acids with a concomitant accumulation of medium-chain fatty acids as observed.

### *pept-1 C. elegans* show increased uptake of free fatty acids from the intestine

**Figure 3:**
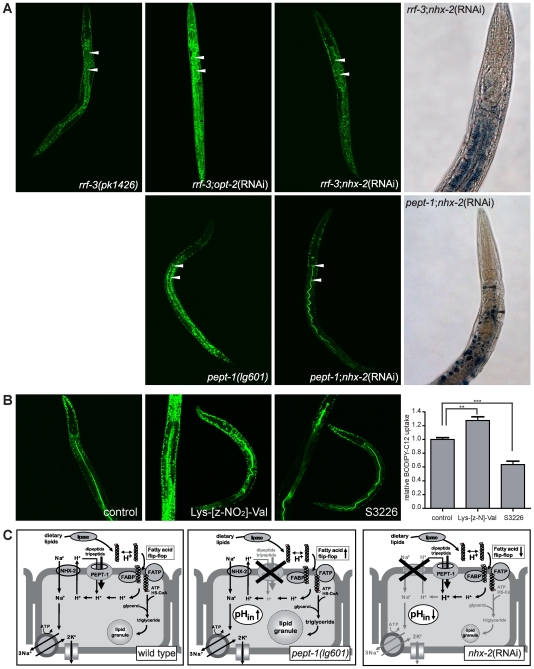
Increased uptake of free fatty acids induces the obesity phenotype in *pept-1(lg601)*. (A) Uptake of free fatty acid BODIPY-C12 in *rrf-3(pk1426)*, *rrf-3;pept-1*(RNAi), *rrf-3;nhx-2*(RNAi), *pept-1(lg601)* and *pept-1;nhx-2*(RNAi) *C. elegans*. *Left panel*, *pept-1(lg601) and rrf-3;pept-1(RNAi)* show a higher uptake of BODIPY-C12 fatty acid indicated by a stronger green fluorescent signal in the cellular fat granules, while *nhx-2* RNAi significantly decreased the uptake of the free fatty acid in *rrf-3* and *pept-1 C. elegans*. A FITC filter was used to visalize BODIPY fluorescence. Arrows indicate the intestinal lumen. *Right panel*, Body fat content in *rrf-3;nhx-2(RNAi)* and *pept-1;nhx-2(RNAi) C. elegans*. *nhx-2* RNAi treatment reduced the body fat in *rrf-3* and *pept-1* worms (compare to Fig. 1A). Body fat was visualized by Sudan Black staining. Arrows indicate single fat granules. (B) Wild type *C. elegans* incubated for one hour in control (M9 buffer containing 0.1% DMSO), 1 mM of the PEPT antagonist Lys-[z-NO_2_]-Val or 1 µM of the NHE3 inhibitor S3226 before BODIPY-C12 free fatty acid uptake. Inhibition of the peptide transporter increases the fatty acid uptake while inhibition of the sodium/proton exchanger reduces fatty acid uptake. The diagram summarizes the relative fluorescent signal intensity, which is directly proportional to the BODIPY-C12 uptake, in the head and anterior intestine of 15–20 individual worms per group. **: p = 0.01, ***: p≤0.001. (C) Proposed model for the uptake of fatty acids into the intestinal epithelial cells of wild type, *pept-1(lg601)* and *nhx-2* RNAi treated *C. elegans*. *Left pane*l, In the intestinal epithelial cells of wild type the PEPT-1-driven proton influx is neutralized by the action of the Na^+^/H^+^ exchanger NHX-2, inducing a neutral intracellular pH. The flip-flop of free fatty acids is happening at a low rate. *Middle panel*, When the peptide transporter is eliminated, the influx of protons is reduced leading to an increased pH_in_. The flip-flop of protonated fatty acids is enhanced and increase desorption of fatty acids into the cell. Higher amounts of triglycerids are formed that are stored in large lipid granules. *Right panel*, The reduced expression of *nhx-2* leads to a PEPT-1-driven slight acidification of the intestinal cells which reduces the flip-flop and therefore the uptake of fatty acids and finally lead to a lean phenotype. *FABP* fatty acid binding protein, *FATP* fatty acid transport protein.

Although there are major alterations in fatty acid metabolism in *pept-1(lg601)* with reduced long-chain FA and PUFA but increased MUFA levels, this does not explain the high fat accumulation in this worm strain. Therefore, we also assessed whether the uptake of free fatty acids from the diet is altered. This was accomplished by the use of a C12 fatty acid that was covalently labeled with the fluorescent dye BODIPY. When fed to worms the BODIPY-C12 fatty acid colocalized with the Nile Red-stained body fat (data not shown), indicating that the fatty acid was taken up by the animals and incorporated into fat stores. However, uptake of the fluorescent fatty acid was much higher in *pept-1(lg601)* and in *rrf-3;pept-1*(RNAi) than in *rrf-3(pk1426)* animals (Fig. 3A). This finding indicates an increased intestinal absorption and storage capacity for fatty acids in peptide transporter deficient worms. The increased uptake of free fatty acid was also detectable in wild type *C. elegans* that were incubated for one hour in 1 mM of the PEPT antagonist Lys-[z-NO_2_]-Val before feeding with BODIPY-C12 (Fig. 3B). In former studies the close functional coupling between di- and tripeptide transport and the Na^+^/H^+^ exchanger NHX-2 in regulation of the pH_in_ was demonstrated [11]. Studies in worms and mammalian intestinal cells [19], [20] have established that apical sodium-proton exchangers maintain intracellular pH homeostasis for proton-coupled nutrient uptake processes including the peptide transporter. Inhibition of sodium-proton exchangers essentially abolishes peptide uptake with a concomitant decline in intracellular pH. One of these processes that is affected by alterations of transmembrane proton gradients is the cellular uptake of free fatty acids via “fatty acid flip-flop” mechanisms [21] (see Discussion and Fig. 3C). We therefore hypothetized that fatty acid uptake into intestinal epithelial cells could be reduced in animals lacking the sodium-proton exchanger NHX-2 while it is increased in those lacking PEPT-1. Indeed, uptake of the BODIPY-C12 fatty acid was drastically reduced by knockdown of *nhx-2* in wild type and *pept-1* animals (Fig. 3A), which lead to a reduced body fat content visualized by Sudan Black stained smaller fat granules in both strains (Fig. 3A). Interestingly, a one hour incubation in 1 µM of the NHE3 inhibitor S3226 (Sanofi-Aventis, Frankfurt, Germany) was also able to decrease the uptake of BODIPY-C12 in wild type *C. elegans* (Fig. 3B). These findings directly prove a close functional link between intracellular pH homeostasis and intestinal fatty acid uptake and storage *in vivo*. Moreover, these findings confirm the data reported by Nehrke, 2003 with a demonstrated lean phenotype in *nhx-2*(RNAi) *C. elegans*.

## Discussion

*C. elegans* with a deletion of the intestinal peptide transporter *pept-1* show markedly increased fat stores with huge fat granules in intestinal cells. Recent work by the Watts group also identified a strongly increased body fat content in *pept-1(lg601)* that is independent on the ingested food bacteria (J. Watts, personal communication). However, these findings are not in line with data presented by Ashrafi et al. (2003) demonstrating a reduced fat content in *pept-1* RNAi treated worms, but discrepancies between knockout and RNAi knockdown phenotype were reported for other genes before. Since lipid accumulation is the product of balance between fat storage and degradation we assessed in *pept-1* all parameters accessible by a combination of analytical and molecular methods. While lipid storage occurs by uptake of fat from the diet or by conversion of an excess of carbohydrates or amino acids via *de novo* synthesis of fatty acids and triaclyglycerols, lipid degradation with release of free fatty acids is followed by β-oxidation in peroxisomes and mitochondria. Long-chain acyl-CoA entering peroxisomes are shortened followed by release of medium-chain fatty acids for complete breakdown in mitochondria and use of acetyl-CoA in the TCA cycle [22]. In *C. elegans* numerous genes encoding enzymes of mitochondrial and peroxisomal β-oxidation are found [23], [24] and we observed for the majority of peroxisomal genes an up-regulation at the transcript level. RNAi knockdown of F08A8.2 and *ech-8* further increased the fat accumulation in the *pept-1* background but not in *rrf-3 C. elegans*. These findings suggest an increased peroxisomal β-oxidation capacity, which may prevent a further lipid accumulation in *pept-1*. The proposed higher flux of long-chain fatty acids through the peroxisomal pathway is supported by the increased mRNA level and activity of peroxisomal catalase that we observed which is needed for detoxification of H_2_O_2_ produced by the acyl-CoA oxidase. The proliferation of peroxisomes is strongly dependent on peroxin proteins. In mammals 13 peroxin genes are known, while the *C. elegans* genome encodes 11 homologues of these proteins [25]. At least four of the dominant peroxin genes display a slightly increased mRNA expression level in *pept-1* when compared to wild type (*prx-5* (+1.25), *prx-6* (+1.1), *prx-11* (+1.83) and *prx-13* (+1.31)). This might suggest an increased peroxisomal proliferation rate in the peptide transporter deficient strain leading to an increased capacity to degrade very long-chain fatty acids. However, levels of medium-chain fatty acids (>C16<C20) were found to be increased in *pept-1*. This could be indicative for a reduced mitochondrial β-oxidation capacity, although transcript profiling did not reveal convincing evidence for altered mitochondrial β-oxidation, as some enzymes showed increased (*acs-3* (+2.3), F58A6.1 (+2.1), B0272.4 (+1.9)) others decreased (*acs-1* (−3.8), *ech-7* (−3.4), F09F7.4 (−2.8)) mRNA levels. Since the medium-chain fatty acids also can be derived from the diet and *pept-1* animals most likely absorb more of these fatty acids, this accumulation of medium-chain fatty acids may also be explained by increased uptake and decreased use for chain elongation and PUFA production.

Using GC- and NMR-based methods, evidence is provided that the fatty acid *de novo* synthesis rate is low in *pept-1(lg601)* and moreover, the animals are deficient in the capacity to produce long-chain and polyunsaturated fatty acids from the dietary precursors. When fed on *E. coli* OP50, the widely used laboratory diet for *C. elegans*, only short and medium-chain fatty acids (C12:0, C14:0 C16:0) are provided by diet whereas long-chain and polyunsaturated fatty acids as well as mmBCFA are not available [18]. As recently shown by NMR approaches, all PUFA's are exclusively synthetized in *C. elegans* [17]. Various enzymes for elongation and desaturation processes are necessary for the synthesis of long-chain fatty acids and their PUFA derivatives. Our data obtained for *pept-1* strongly suggest that animals have an impaired capacity for synthesis of these fatty acids. This is mirrored by low transcript levels of the enzymes involved and low values of ^13^C_2_-isotopologues in long-chain FAs, as observed in the *in vivo* feeding experiment using ^13^C-labeled *E. coli*. Notably, only the relative fraction of isotopologues in long-chain FA due to uptake (i.e. [010+111]) and due to *de novo* synthesis (i.e. [011+110]) is presented. Therefore, the absolute rates of uptake or *de novo* fatty acid synthesis can not be calculated on this basis. Nevertheless, the increased levels of medium-chain fatty acids such as C16:1Δ9 with 2.5-fold and C18:1Δ9 with 1.4-fold increased level suggest that these two most important precursor fatty acids for elongation processes in the worm [26] are taken up from the diet with increased capacity.

Absorption of fatty acids from the gut lumen has been rarely studied in *C. elegans*. Although the worm genome contains several genes coding for proteins with homology to intestinal lipases, fatty acid transporter proteins (FATP) and fatty acid binding proteins (FABP) it is yet unclear if and to which extent triglycerides are degraded in the gut lumen and how free fatty acids are taken up into the intestinal epithelial cells [13]. Interestingly, the gene F46B6.8 that codes for a structural homologue of the mammalian gastric triacylglyceride lipase (EC 3.1.1.3) showed a 13-fold increased mRNA level in *pept-1* when compared to wild type (Table S1 available online). When we applied RNAi for F46B6.8 no detectable changes in cell morphology or development of *pept-1* worms were observed but average fat droplet size in intestinal cells was reduced (data not shown). Assuming that increased mRNA levels of this lipase translate into increased enzymatic activity, the capacity of triglyceride degradation in the gut lumen could be increased resulting in the accelerated release of free fatty acids followed by uptake into intestinal epithelial cells. We did observe that fatty acid uptake and incorporation into intestinal lipid droplets – probed with a fluorescent fatty acid derivative – is markedly increased in *pept-1*. However, so far it is not known how fatty acid uptake is achieved in intestinal cells and which proteins are involved in *C. elegans*.

Fatty acid uptake into cells generally involves fatty acid transporters as integral membrane proteins (FATP) and fatty acid binding proteins (FABP) [27], [28]. There is a controversy on whether the FATPs are solely mediating fatty acid permeation through the cell membrane or possess additionally catalytic activity as acyl-CoA synthetases. As the genome of *C. elegans* contains homologous genes for most of these transport and binding proteins, it is predicted that these mechanisms are also conserved in the nematode [13]. Regardless of putative proteins that may allow increased fatty acid uptake, unesterified fatty acids also cross cell membranes in their protonated and therefore lipophilic form by the so called flip-flop mechanism [21]. Here initially the fatty acid adsorbes from the lumen into the outer leaflet of the plasma membrane, it then crosses the membrane with a re-orientation of the carboxylic group to the cytosolic site followed by deprotonation and finally the anion leaves the cytosolic leaflet for binding to fatty acid binding proteins or acyl-CoA-synthetase in the cytoplasm. Fatty acid permeation into the cell is consequently associated with intracellular acidification and this has already been shown in various cell types [29], [30], [31]. The fatty acid flip-flop rate and therefore fatty acid uptake via this biophysical process is increased when the intracellular pH is less acidic than the pH_out_.

Since PEPT-1, like its mammalian counterpart PEPT1, co-transports protons into the cell when di- or tripeptides are taken up, PEPT-1 acts as an acid loader and causes a decrease in intracellular pH. The protons are re-exported by the Na^+^/H^+^ exchanger NHX-2 to the luminal side. The imported sodium ions leave the cell in exchange with potassium ions via the sodium potassium ATPase in the basolateral side of the cells. In the absence of the peptide transporter, proton influx into epithelial cells is low and the intracellular pH increases which in turn promote the flip-flop of fatty acids over the cell membrane. This would enable enhanced uptake of free fatty acids and that we clearly demonstrate to be the case with the use of the fluorescent fatty acid derivative in *pept-1* and in wild type *C. elegans* treated with the PEPT antagonist Lys-[z-NO_2_]-Val (Fig. 3A and B). Inactivation of the Na^+^/H^+^ exchanger NHX-2 should cause the opposite; a reduced fatty acid import and that is what we observe as shown in Fig. 3 when NHX-2 expression is down-regulated by RNAi or when it is blocked by the specific inhibitor S3226. Moreover, these animals are extremely lean also previously observed in *C. elegans* lacking *nhx-2* [11]. We could show that the effect is also dominant in the *pept-1* background, where nhx-2(RNAi) significantly reduced the fat granule size/fat content (Fig. 3A). The mechanisms underlying this fatty acid transport regulation based on the cooperation of peptide transporters and Na^+^/H^+^ exchangers involving alterations in pH_in_ are displayed in the proposed model provided in Fig. 3C. Our data strongly suggest that the increased fat storage in *pept-1* animals is a consequence of this increased fatty acid uptake into intestinal cells promoted by the lack of appropriate intracellular acidification rates during digestion and absorption in the absence of the peptide transporter. In summary, the regulation of the intracellular pH in intestinal epithelial cells seems to be key regulator of the uptake of fatty acids from the diet, which is the central process for body fat homeostasis in obese *pept-1* and lean *nhx-2* animals.

We here provide evidence for a central mechanism by which apical membrane transporters for di- and tripeptides and sodium-proton exchange in intestinal epithelial cells contribute to fatty acid and lipid homeostasis in *C. elegans*, whereas *pept-1(lg601)* animals are obese, animals lacking *nhx-2* are lean. The interplay of the two intestinal proteins in regulation of intracellular pH seems of fundamental importance for the capacity of fatty acid uptake from the gut lumen and their storage in lipid granules. In addition, animals lacking *pept-1* have a reduced capacity of *de novo* fatty acid synthesis, a reduced synthesis of PUFA and possibly an increased β-oxidation of long-chain fatty acids which all may be driven by the obese phenotype.

## Materials and Methods

### *C. elegans* strains and nematode culture

The following *C. elegans* strains were used in this study: wild type N2 var. Bristol, *rrf-3(pk1426)*II, *daf-2(e1370)*III, *pept-1(lg601)*X (formally *pep-2(lg601)X*) [5]. Wild type, *rrf-3* and *daf-2* were received from the *C. elegans* Genetics Center (CGC, Minneapolis, USA). The worms were grown at 20°C on Nematode Growth Medium (NGM) agar plates with *E. coli* bacteria OP50 as food source [14]. A mixed-stage worm culture was washed of the plates with M9 buffer, eggs were prepared by hypochlorite treatment and were allowed to hatch over night in M9 buffer. The synchronised L1 larvae were grown on NGM agar plates with *E. coli* OP50 till the fourth larval state (L4). The L4 larvae were harvested and washed five to six times in M9 buffer to minimize bacteria contamination. Aliquots of 150 µl worms in M9 buffer were frozen in liquid nitrogen and stored at −80°C.

### RNA preparation and microarray analysis

Total RNA was isolated from each *C. elegans* sample using a combination of TRIZOL® (Invitrogen, Karlsruhe, Germany) till the ethanol precipitation step followed by purification via the RNeasy Mini Kit (Qiagen, Hilden, Germany). Total RNA was reverse transcribed and the corresponding cRNA was biotinylated and fragmented following the original protocol of Affymetrix (Affymetrix Inc., Santa Clara, CA, USA). For each *C. elegans* strain five independent cRNA samples were hybridized over night on Affymetrix *C. elegans* whole-genome arrays and the arrays were washed and scanned following the instructions of the provider.

### Transcriptome data analysis and statistics

The quality of the data was analysed by a bioconductor and R based method in the Nutrigenomics Organisation NuGO Array Pipeline. To calculate gene expression measures, the preprocessing method GCRMA was used [32], which is implemented in the package ‘gcrma’ of bioconductor (www.bioconductor.org). Bioconductor is an open source software project for the analysis and comprehension of genomic data based on a free statistical programming language R. Three procedures for preprocessing were employed: background adjustment with GCRMA, normalization with the Quantile method, and finally summarization with median polish procedure. After preprocessing, significance analysis of microarrays (SAM) [33], which is implemented in the package ‘siggene’ of bioconductor, was performed on different mutant strains comparing with the wild type to identify the genes with significant changes on expression levels. For different strains, different thresholds were applied according to the ‘elbow heuristic’, which is trying to minimize the false discovery rate with a comparably large ‘delta’. To interpret the selected genes biologically, the information from Gene Ontology (www.geneontology.org), and Wormbase (www.wormbase.org) was employed to annotate these genes.

### RNAi feeding

The *nhx-2*(RNAi) construct as well as the others (F08A8.2, *ech-8* and *pept-1*) are from the Ahringer *C. elegans* RNAi library [34] and were used as described previously [35]. As a control for RNAi experiments, nematodes were grown on NGM agar plates with the HT115 *E. coli* transformed with pPD129.36 (L4440) empty plasmid for at least two generations.

### Fat staining

Sudan Black staining was done according to an established protocol [36]. For visualisation of the black-blue stained fat granules we used a Leica DM IRB microscope (Leica, Wetzlar, Germany) with a digital camera.

### Fat extraction and gas chromatographical analysis of fatty acids

For biochemical analysis, lipids were extracted from 130–170 mg of synchronised L4 larvae with chloroform-methanol (1∶1) by volume [37]. The hydrophilic portion of each sample was lyophilized (Alpha 1–4 LD plus, Christ, Germany) and the fatty acid concentration was calculated in relation to the dry weight of the sample. The individual fatty acids in the lipid fractions were transesterified to fatty acid methyl esters as described by [38]. The fatty acid methyl ester concentrations were determined using fused silica capillary column BD23 (Agilent Technologies) fitted in a gas chromatograph (HP 6890, Agilent Technologies). Hydrogen was used as carrier gas.

### ^13^C-labelling of *C. elegans* with [U-^13^C]-*E. coli* cells

*E. coli* M15 [pREP4, pRFN4] strain [39] was grown on M9 minimal medium supplemented with vitamins, trace elements, ampicillin (50 mg/l) as well as kanamycin (10 mg/l) and containing 3 g/l [U-^13^C_6_]glucose (>99% abundance; from Cambridge Isotop Laboratories (Andover, MA, USA)) as the single source of carbon and energy. The cells were harvested in early stationary phase, washed with solution containing 0.9% saline, 15% glycerol and stored at −80°C. Mixed populations of wild type *C. elegans* were grown on NGM agar plates with *E. coli* OP50 as food source [14]. The nematodes (3–4g) were transferred to NGM agar plates with ^13^C-enriched bacteria (mixture of 10% [U-^13^C]-*E. coli* M15 and 90% unlabeled *E. coli* M15) and were cultured on these bacteria for 15 hours at 15°C. Nematodes were harvested, washed several times with M9 buffer to avoid bacteria contamination, frozen in liquid nitrogen and stored at −80°C.

### Lipid extraction and NMR analysis

The frozen worms were lyophilized and weighed. They were then extracted with 40 ml of 50% aqueous methanol at room temperature for 2 h. The remaining biomass was extracted with 15 ml of dichloromethane at 45°C under reflux. The extract was dried under reduced pressure. The residue was dissolved in 500 µl of CDCl_3_ and analysed by NMR spectroscropy. ^13^C-NMR spectra were recorded at 25°C using a DRX 500 spectrometer (Bruker Instruments, Karlsruhe, Germany) at a transmitter frequency of 125.6 MHz. ^13^C-coupled satellites of a given signal group were integrated separately. The relative fractions of each respective satellite pair (corresponding to a given coupling pattern) in the total signal integral of a given carbon atom or an atom group were calculated.

### Labelling of *C. elegans* with a fluorescent fatty acid probe

To determine time-dependent fatty acid uptake in *C. elegans*, L4 larvae were labelled with the fatty acid analog 4,4-difluoro-5-methyl-4-bora-3a,4a-diaza-3-indacene-dodecanoic acid (BODIPY 500/510 C1,C12, Invitrogen, Molecular Probes). BODIPY-C12 was dissolved in 100% DMSO to a stock concentration of 2.4 µmol/l. L4 larvae were incubated for 10 minutes in M9 buffer containing a final concentration of 20 nmol/l BODIPY-C12 (and 0.1% DMSO). Worms were washed several times in M9 buffer before loading to an object slide. The BODIPY fluorescence was visualized using a Leica TCS SP2 Confocal System coupled to a DM IRB microscope (Leica, Wetzlar, Germany). To test the impact of PEPT and NHX-2 inhibitors on fatty acid uptake, wild type *C. elegans* were incubated for one hour in M9 buffer containing 1 mM of the PEPT antagonist Lys-[z-NO_2_]-Val (solved in water; z-NO2: 4-nitrobenzyloxycarbonyl; provided by Prof. K. Neubert, Halle, Germany), 1 µM of the NHE3 inhibitor S3226 (solved in DMSO; provided by Sanofi-Aventis) or in M9 buffer containing 0.1% DMSO as control.

### Accession numbers

The GenBank accession numbers (http:// www.ncbi.nlm.nih.gov/Genbank/) for genes used in this study are: *pept-1* (NM_076686), *daf-2* (NM_001129262), *rrf-3* (NM_063312), *nhx-2* (NM_063213), *ech-8* (NM_069475), F08A8.2 (NM_060861).

## Supporting Information

Table S1 Regulated genes in pept-1. Table of all significant regulated gene transcripts in pept-1 when compared to wild type C. elegans. Results are from an Affymetrix microarray experiment.
(0.50 MB XLS)
